# Water-soluble Fraction of *Abelmoschus esculentus* L Interacts with Glucose and Metformin Hydrochloride and Alters Their Absorption Kinetics after Coadministration in Rats

**DOI:** 10.5402/2011/260537

**Published:** 2011-09-11

**Authors:** Hajera Khatun, Ajijur Rahman, Mohitosh Biswas, Anwar Ul Islam

**Affiliations:** ^1^Department of Pharmacy, Southeast University, Dhaka 1213, Bangladesh; ^2^Department of Pharmacy, University of Rajshahi, Rajshahi 6205, Bangladesh; ^3^Department of Pharmacy, University of Asia Pacific, Dhaka 1209, Bangladesh

## Abstract

This study was done to investigate the effects of water-soluble fraction (WSF) of the fruits of *Abelmoschus esculentus* L (okra/lady's fingers) on absorption of oral glucose as well as metformin from the gastrointestinal tract in the Long Evans rats. WSF of *A. esculentus* significantly (*P* < 0.05) reduced the absorption of glucose as studied in the 24 hrs fasting rats. The effect of WSF of *A. esculentus* on metformin absorption was studied in alloxan-induced diabetic rats. Significant differences (*P* < 0.05) were observed in the average blood glucose level from 2 to 24 hours after metformin therapy in presence (33.6 to 34.2 mmol/L) or absence (15.2 to 20.2 mmol/L) of oral WSF of *A. esculentus*. In both of the experiments, Na-carboxymethylcellulose (CMC) was used as positive control. The results of this study indicate that *A. esculentus* may improve glycemic control but should not be taken concurrently with metformin hydrochloride in controlling diabetes mellitus.

## 1. Introduction

Diabetes mellitus (DM) is one of the most common problems challenging the public health in the 21st century [1]. It is a metabolic disorder marked by high blood sugar level (hyperglycemia), occurs when pancreas cannot produce enough of insulin (type 1 DM) or body cannot effectively use the produced insulin (type 2 DM). According to WHO, more than 220 million people worldwide are suffering from DM and 90 to 95% are of type 2 DM. In 2004, about 3.4 million people died from diabetes [2]. Treatment measures of DM include diet control, physical exercise, and use of oral medications for type 2 only or use of insulin in type 1 DM. Recently, the use of high-fiber diets, especially of the soluble variety, and soluble fiber supplements in controlling DM is being discussed extensively. Water-soluble dietary fibers have the potential to reduce glucose absorption, increase in hepatic extraction of insulin, increased insulin sensitivity at the cellular level, and binding of bile acids [3].

Metformin, a biguanide class antihyperglycemic agent, is widely used to treat type 2 DM. It helps to control the amount of blood glucose by decreasing glucose absorption from the gastrointestinal tract and formation of glucose by the liver. Metformin also increases body's response to insulin [4]. It is taken orally with meals to reduce its strong gastrointestinal side effects like diarrhea, cramps, nausea, vomiting, and increased flatulence [5]. As it is taken with meals, there is a chance of interaction with foods which may alter the absorption kinetics of it. Interaction of metformin with guar gum, a dietary fiber has been studied in human beings and it was found that guar gum reduced the absorption of metformin over the first 6 hrs [6]. On the other hand, it is suggested using dietary fibers and some vegetable extracts to reduce postprandial hyperglycemia. So, it is matter of concern whether metformin is safe to take with a meal that contains any source of soluble dietary fiber (SDF).

The green fruits of okra/lady's fingers (*Abelmoschus esculentus *L) are popular all over the world as a vegetable for its nutritional values and health benefits [7]. Okra is a flowering plant cultivated in tropical, subtropical, and warm temperate regions around the world [8]. The pods are full of nutrients like minerals, vitamins, proteins, carbohydrates, fats, enzymes and large amount of mucilage which contains soluble dietary fibers like pectin, guar gum, carboxymethylcellulose (CMC), and so forth [9]. Traditionally, okra has been used as an alternative treatment for diabetes [10]. It is assumed that this effect of okra is due to the presence of large amount of soluble dietary fibers which retard glucose absorption from the gastrointestinal tract. Thus, the possible interaction of the soluble dietary fiber fraction of okra with oral metformin is a matter of concern because this vegetable is being widely used by the diabetics as an adjunction to the DM treatment.

This report describes herein, the effect of WSF of okra on glucose absorption in fasting rats as well as the absorption kinetics of metformin in alloxan-induced diabetic rats. So far we know this is first scientific report about beneficial effect of okra in reducing postprandial serum glucose and interaction of WSF of okra with metformin which results in loss of antihyperglycemic activity of this drug.

## 2. Materials and Methods

### 2.1. Preparation of Viscous Water-soluble Fraction of *Abelmoschus esculentus* L

500 gm of fresh pods or fruits of *Abelmoschus esculentus *L were collected in the month of May, 2009 from a local market of Rajshahi, Bangladesh. Then, the pods were thoroughly washed with distilled water, cut into small slices by a sharp knife. About 45 gm of the sliced pods were immersed into 150 mL distilled water in a beaker. The mixture was then stirred gently for 10 to 15 minutes with a glass rod; filtered using a thin layer of cotton to remove the insoluble matters and filtrate was collected. The amount of filtrate was measured and the amount of dried constituents per milliliter was also measured. Viscosity of the solution was measured using Brookfield viscometer and found to be 50 cP  (mPa.s.).

### 2.2. Experimental Animals

Experiments were performed on a total of 60 Long Evans rats weighing about 80–100 gm of either sex, purchased from ICDDRB, Dhaka, Bangladesh. They were maintained in colony cages (3 rats per cage) at an ambient temperature of 25–27°C with 12-hours light and dark cycles having proper ventilation in the room. They were fed normal diets purchased commercially from the vendors and water *ad libitum*. The rats were allowed to acclimatize to the laboratory environment for one week and were randomly divided into the groups for experiments.

### 2.3. Chemicals, Drugs, and Instruments

Metformin (Sigma) was donated by Chemico Laboratories, Rajshahi. Glucose (GlaxoSmithKline), Glucometer (Bioland, Germany), alloxan (Sigma), and CMC (Fluka, Switzerland, dynamic viscosity 1000 mPa.s in 2% w/v water solution, pH 6.5–8.0.) were purchased from local suppliers.

### 2.4. Induction of Diabetes

Diabetes was induced in rats by a single intravenous injection of alloxan (120 mg/kg, b.w.). Five days later, the plasma glucose concentration was determined in blood samples obtained from rats. Rats that were not diabetic (<14.7 mmol/L) or that were extremely diabetic (>35.5 mmol/L) were excluded from the study.

### 2.5. Experimental Design and Administration of Doses

Five groups of rats (*n* = 6 per group) were used to study the effect of WSF on glucose absorption: normal control (NC), fasting control (FC), fasting with glucose only (FG), fasting with glucose, and WSF of *A. esculentus* (FGF), fasting with glucose and CMC (positive control, FGC). The rats of all groups except the NC group were fasted for 24 hours and fasting blood glucose levels were determined. Then, 0.1 mL of glucose (2 gm/kg), mixture of glucose-WSF of *Abelmoschus esculentus* L (0.1 mL glucose with 0.2 mL WSF of *Abelmoschus esculentus*) and mixture of glucose-CMC (0.1 mL glucose with 0.2 mL CMC; 1% w/v) were administered orally to the respective groups of rats by gastric feeding tube, whereas fasting control group received only the vehicle. Normal control rats were fed standard diets.

To investigate the effects of WSF of *A. esculentus* L on metformin absorption another 30 Long Evans rats were randomly divided in one of five groups (*n* = 6 per group): normal control (NC), diabetic control (DC), diabetic with metformin only (metformin control, MC), diabetic with metformin and WSF of *A. esculentus* (DMF) and diabetic with metformin and CMC (positive control, DMC). Metformin was dissolved in sterile distilled water. Dosing mixtures of metformin + WSF of *A. esculentus* and metformin + CMC were prepared by mixing 0.2 mL of WSF (0.34 gm/mL) or CMC with 0.1 mL of metformin in a concentration providing 200 mg/kg body weight of rats. Dosing mixtures and metformin only solutions were orally administered to the alloxan-induced diabetic rats by gastric feeding tube whereas the control groups received only the vehicle.

Blood was taken from the rats by tail snipping at 2, 4, 8, 12, and 24 hours after the treatment and blood glucose was estimated using glucose meter (Bioland, Germany).

### 2.6. Statistical Analysis

Significance of differences between the mean values were determined by analysis of variance (ANOVA), followed by Dunnett's test. SPSS 15.0, USA were used for statistical analysis. Graphs were prepared by using Microsoft-Excel version 2003. Results were considered significant when the *P* values were less than 0.05.

## 3. Results and Discussion

### 3.1. Effect of WSF of *A. esculentus* on Glucose Absorption in Fasting Rats

**Figure 1:**
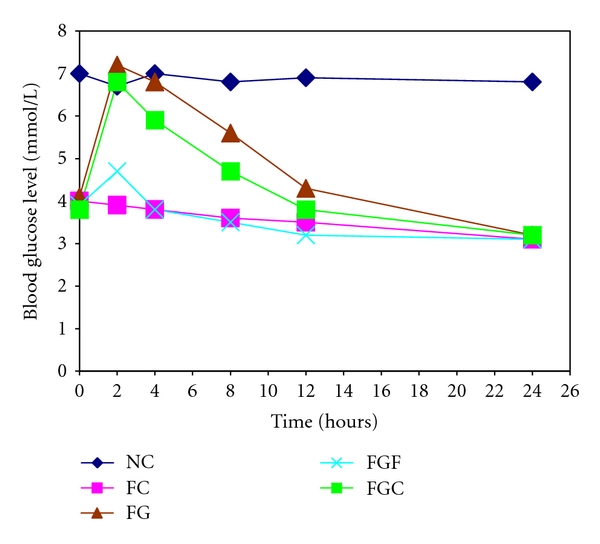
Blood glucose level in 24 hours fasted rats after different treatments. NC: normal control; FC: fasting control; FG: fasting glucose; FGF: fasting with glucose and WSF of *A*. *esculentus* and FGC, fasting with glucose and CMC.

As shown in Table 1 and Figure 1, WSF of *A. esculentus* significantly reduces the absorption of glucose. Five groups of rats (*n* = 6 for each group) were used for this study. The rats other than the normal control were kept in fasting condition for 24 hours to eliminate the relevant gastrointestinal factors. Glucose (2 gm/kg b.w), WSF of okra (0.2 mL) and CMC (0.2 mL) were administered according to the experimental design and blood glucose was measured at different time intervals (Table 1). We found that WSF of *A*.* esculentus* significantly (*P* < 0.05 compared to FG group) reduced the absorption of glucose which is reflected in the blood glucose level (Table 1, Figure 1). At 2 hrs, the average blood glucose level in the rats that received only glucose was 7.2 mmol/L but when the same amount of glucose was administered with 0.2 mL of WSF in FGF group rats, the level reduced to 4.7 mmol/L. The effect of okra was significantly higher than of CMC (positive control). This effect of WSF of okra and purified CMC corresponds to the previously reported *in vitro* experiments by us [11]. 

Previous studies reported that consumption of viscous water-soluble dietary fibers reduced postprandial blood glucose by reducing the diffusion of glucose and postponing the absorption and digestion of carbohydrates [12]. It has also been reported that different types of dietary fibers (especially soluble) reduced the diffusion of glucose* in vitro* [13] and also *in vivo* [14, 15]. Our data are consistent with these earlier studies.

### 3.2. Effect of WSF of *A. esculentus* on Metformin Absorption in Allloxan-Induced Diabetic Rats

**Figure 2:**
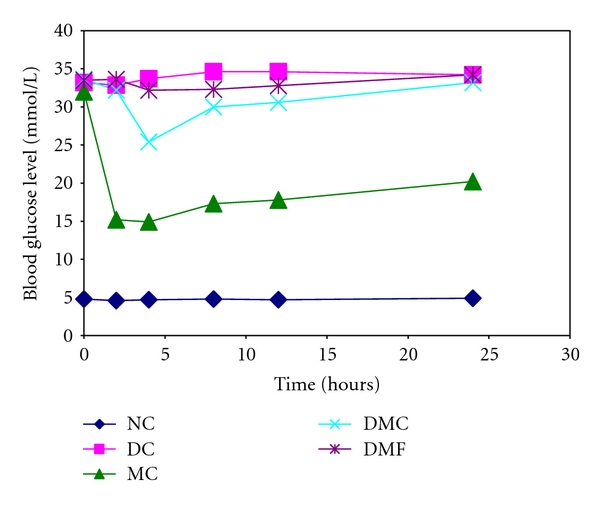
Blood glucose level in alloxan-induced diabetic rats after oral administration of metformin alone or with WSF of lady's fingers and CMC. NC: normal control; DC: diabetic control; MC: diabetic with metformin only (metformin control); DMF: diabetic with metformin and WSF of *A. esculentus*; DMC: diabetic with metformin and CMC.

We have also seen a strong interaction between WSF of okra and metformin which results in nearly complete loss of antihyperglycemic effect of metformin. Alloxan-induced diabetic rats were used in this study. Metformin (200 mg/kg b.w.) was administered in solution form to the rats alone or with WSF of okra or CMC whereas normal control and diabetic control group were fed only normal diet. Glucose level in blood was then measured with a glucose meter at different time intervals (Table 2). When metformin was administered alone, the blood glucose level of the diabetic rats was reduced from 32.0 to 14.9 mmol/L within 4 hours. After coadministration of metformin with WSF of lady's fingers in the DMF group of rats, surprisingly the effect of metformin is almost lost. Blood glucose level changed very little in this group of rats (33.5 to 32.2 mmol/L at 4 hrs). But, in the animals which did not receive WSF of okra with metformin, glucose level dropped to 14.9 mmol/L within 4 hours. CMC also exhibited the same effect like WSF of lady's fingers. The glucose level decreased from 33.6 to 25.4 mmol/L when metformin was given with CMC (Table 2 and Figure 2). But, CMC may have released small amount of metformin which reduced the blood glucose level to 25.4 mmol/L by 4 hrs whereas the effect of WSF of okra was consistent and it released very little of metformin over the period of 24 hrs. These observations can be explained by the fact that WSF of okra and CMC entrapped metformin, thus made it unavailable for absorption. The molecular interaction between the amine group of metformin and carboxyl groups of the components of WSF may be the reason of this entrapment. High viscosity of WSF of okra (50 cP) may also contribute to this effect by inhibiting the diffusion of metformin from the dosing mixtures [13]. Now we are working to discover the exact mechanism by which glucose and metformin absorption was altered by WSF of okra. More study should be done whether there are any other factors other than the entrapment of metformin by the fiber contents of okra are responsible for this effect.

The result of our study complies with the study performed previously by Gin et al. [6]. They studied the effect of guar gum on the digestive absorption of metformin in 6 healthy subjects and found that when given together with guar gum there was a reduction in the absorption rate of metformin over the first 6 hours. In our study we have observed that the effect of WSF of okra in reducing the absorption is more stable than that of guar gum. This may be due to the presence of a mixture of soluble fibers in WSF of okra. The presence of large amount of carbohydrates (7 gm%) in okra may also contribute to the entrapment of metformin.

## 4. Conclusion

Use of dietary fiber has been correlated with the prevention of many life-threatening diseases like diabetes, heart disease, cancers and so forth. Okra (*Abelmoschus esculentus* L.) is a rich source of dietary fibers and is traditionally used in the management of diabetes mellitus. Moreover, hypolipidemic effect of *Abelmoschus esculentus *L has been reported which also benefits the diabetic patients [16, 17]. In this study, we also found that viscous soluble dietary fiber of *Abelmoschus esculentus* L. significantly reduced the intestinal absorption of glucose in fasting rats. So, okra may be beneficial for diabetic patients to control the postprandial blood glucose level. But, we also observed that both CMC and WSF of okra significantly reduced the absorption of metformin *in vivo*. These observations suggest that type 2 DM patients should be careful in taking metformin with a meal that contains lady's fingers. Further studies are required to elucidate the effects of other fiber-containing foods on the effect of antidiabetic drugs. This study was done in experimental rats. To properly interpret these interesting findings, the study should be done using human subjects.

## Figures and Tables

**Table 1:** Blood glucose level (mmol/L) after oral administration of glucose, WSF of okra, and CMC.

Treatment groups	0 hr	2 hrs	4 hrs	8 hrs	12 hrs	24 hrs
NC	7.0 ± 0.02	6.7 ± 0.12	7.0 ± 0.19	6.8 ± 0.04	6.9 ± 0.05	6.8 ± 0.13
FC	4.0 ± 0.03	3.9 ± 1.05	3.8 ± 0.05	3.6 ± 0.05	3.5 ± 0.06	3.1 ± 0.05
FG	4.1 ± 0.10	7.2 ± 0.10	6.8 ± 0.02	5.6.±1.02	4.3.±1.00	3.2 ± 0.03
FGF	3.9 ± 0.12	4.7 ± 0.23^a,b^	3.8 ± 1.24^a,b^	3.5 ± 0.24	3.2 ± 0.40	3.1 ± 0.19
FGC	3.8 ± 1.04	6.8 ± 0.95	5.9 ± 1.35	4.7 ± 2.04	3.8 ± 0.90	3.2 ± 1.07

Values are expressed as mean ± SEM of 6 rats/group. NC: normal control; FC: fasting control; FG: fasting with glucose (2 gm/kg b.w.); FGF: fasting with glucose (2 gm/kg b.w.) and WSF of *A. esculentus* (2 gm/kg b.w.); and FGC: fasting with glucose and CMC. ^a^ *P* < 0.05 compared to FG; ^b^ *P* < 0.05 compared to FGC (ANOVA followed by Dunnett's test).

**Table 2:** Blood glucose level (mmol/L) in alloxan-induced diabetic rats after oral administration of metformin, WSF of okra and CMC in alloxan-induced diabetic rats.

Treatment groups	0 hr	2 hrs	4 hrs	8 hrs	12 hrs	24 hrs
NC	4.8 ± 0.04	4.6 ± 0.20	4.7 ± 0.19	4.78 ± 0.02	4.69 ± 0.15	4.9 ± 0.03
DC	33.2 ± 1.02	32.85 ± 0.14	33.7 ± 1.01	34.6 ± 1.35	34.6 ± 0.06	34.2 ± 1.05
MC	32.0 ± 0.40	15.2 ± 0.09	14.9 ± 1.0	17.3 ± 0.04	17.8 ± 1.90	20.2 ± 1.01
DMF	33.5 ± 1.05	33.6 ± 0.95^a^	32.2 ± 1.35^a^	32.3 ± 2.04^a^	32.8 ± 1.02^a^	34.2 ± 1.07^a^
DMC	33.6 ± 0.19	32.2 ± 0.40^a^	25.4 ± 0.1^a^	30.0 ± 1.24^a^	30.6 ± 0.14^a^	33.2 ± 0.09^a^

Values are expressed as mean ± SEM of 6 rats/group. NC: normal control; DC: diabetic control; MC: diabetic with metformin only (metformin control); DMF: diabetic with metformin and WSF of *A. esculentus*; DMC: diabetic with metformin and CMC. ^a^ *P* < 0.05 compared to MC (ANOVA followed by Dunnett's test).

## Abbreviations

CMC: Na-carboxymethylcellulose
DC: Diabetic control
DM: Diabetic mellitus
DMC: Diabetic with metformin and CMC
DMF: Diabetic with metformin and WSF of *A. esculentus *
FC: Fasting control
FG: Fasting + glucose
FGC: Fasting with glucose and CMC
FGF: Fasting with glucose and WSF of *A. esculentus *
MC: Diabetic with metformin only (metformin control)
NC: Normal control rats
SDF: Soluble dietary fiber
WSF: Water-soluble fraction.
